# Interactive effect between tree ageing and trunk-boring pest reduces hydraulics and carbon metabolism in *Hippophae rhamnoides*

**DOI:** 10.1093/aobpla/plac051

**Published:** 2022-10-22

**Authors:** Lin Wang, Junpeng Li, Yang Wang, Hao Xue, Yongxin Dai, Youzhi Han

**Affiliations:** College of Forestry, Shanxi Agricultural University, Taigu, Shanxi 030801, P.R. China; College of Forestry, Shanxi Agricultural University, Taigu, Shanxi 030801, P.R. China; College of Forestry, Shanxi Agricultural University, Taigu, Shanxi 030801, P.R. China; College of Forestry, Shanxi Agricultural University, Taigu, Shanxi 030801, P.R. China; College of Forestry, Shanxi Agricultural University, Taigu, Shanxi 030801, P.R. China; College of Forestry, Shanxi Agricultural University, Taigu, Shanxi 030801, P.R. China

**Keywords:** Carbon metabolism, hydraulic characteristic, *Holcocerus hippophaecolus* damage, sea-buckthorn (*Hippophae rhamnoides*) decline, Tree ageing

## Abstract

Sea-buckthorn (*Hippophae rhamnoides*) is widely distributed across the Eurasian continent. Recently sea-buckthorn has shown premature ageing and decline when confronted with water deficiency and *Holcocerus hippophaecolus* damage in northwest China and the Loess Plateau region. However, the physiological process of sea-buckthorn senescence in response to drought and pest damage is still unknown. In this study, 4-year-old (4y), 15-year-old normal growth (15yN) and 15-year-old seriously moth-damaged sea-buckthorn plants (15yH) were used as the research objects. The growth of branches and roots, branch water potential and percentage loss of conductivity (PLC), branch vulnerability to embolism (quantified by P_50_, xylem water potential at 50 % of PLC), branch xylem parenchyma cell viability, photosynthesis and the non-structural carbohydrate (NSC) content in branches and roots in dry and wet seasons were measured. The results showed that the length, basal diameter of 1-year-old branches and the leaf area of 4y trees were significantly larger than that of 15yN and 15yH trees, and the fine root density of 15yH trees was significantly lower than that of 15yN trees in all measured areas. The branch-specific hydraulic conductivity of 15yN and 15yH trees was only 50.2 % and 12.3 % of that of 4y trees, and the P_50_ of 4y, 15yH and 15yN trees was −3.69 MPa, −2.71 MPa and −1.15 MPa, respectively. The midday water potential and photosynthetic rate were highest in 4y trees, followed by 15yN and then 15yH trees in both the dry season and wet seasons, while branch PLC declined in the opposite direction (15yH trees highest, 4y trees lowest). The degree of PLC repair within a day was highest in 4y trees, followed by 15yN and then 15yH trees, and the viability of xylem cells was consistent with this pattern. The branch xylem starch and NSC content of 4y and 15yN trees were significantly higher than that of 15yH trees in the dry season, and the root starch and NSC content of 4y trees were significantly higher than that of 15yH trees in the two seasons. The above results suggest that the hydraulic properties of the normal elderly and seriously pest-damaged sea-buckthorn were significantly worse than in juvenile plants. Narrower early wood width and vessel density, high embolism vulnerability and weak embolism repair capacity led to the decline in water-conducting ability, and similarly further affected photosynthesis and the root NSC content. The decline in xylem parenchyma cell viability was the main reason for the limited embolism repair in the branches.

## Teaser

Both ageing and insect damage by *Holcocerus hippophaecolus*led to the reduction of hydraulic efficiency, hydraulic safety and xylem embolism repair capacity of aged sea-buckthorn, which constrained the photosynthesis and root carbon storage. Insect damage further deteriorated water status and carbon shortage, and similarly limited root growth and weakened the trees. Also, the capacity of xylem embolism repair was closely related to the cell viability of branches. Results indicated that poor water status and reduced carbon supply in roots would affect the survival of aged and moth-damaged sea-buckthorn under adverse conditions.

## Introduction

Sea-buckthorn (*Hippophae rhamnoides*) is widely distributed in the northwest, southwest and northeast of China. It has good environmental adaptability and high nitrogen-fixation ability, so it has attracted extensive attention as an economic forest species and a species for water and soil conservation (Lian and Chen 1996). In recent years, sea-buckthorn decline has become a problem, especially in plantations. Previous studies have shown that frequent drought in China’s north-western region has resulted in a decrease in sea-buckthorn growth, early ageing and even death (Tang *et al.* 2014; Cao *et al.* 2016). Moreover, some studies have reported that *Holcocerus hippophaecolus* damage to the trunk and main roots was an important cause of sea-buckthorn decay (Zong *et al.* 2011; Xu *et al.* 2018). *Holcocerus hippophaecolus* damage mainly starts from the stem base, concentrated at the root collar, then destroys the xylem and phloem of trees, affecting water transport and photosynthetic product transport. In addition, ageing trees may be confronted with an inadequate water supply and insufficient carbon supply (Lindenmayer *et al.* 2012; Rowland *et al.* 2015; Hammond and Adams 2019). However, the effects of ageing, trunk borer pest damage and their interaction under seasonal drought conditions are still unknown.

Water balance and carbon metabolism are the basis for tree growth and survival (McDowell 2011; Rowland *et al.* 2015; Dai *et al.* 2018). Water shortage could cause a decline in the water potential and an increase in the tension of xylem sap; the large negative pressure that results may lead to embolism (Tyree 2003). Severe embolism can destroy the integrity of water-conducting paths and limit water transport from roots to leaves, leading to hydraulic failure, which is the main mechanism of drought-induced tree mortality (Choat *et al.* 2018; Hammond *et al.* 2019). Another important cause of drought-induced tree mortality is carbon starvation, which limits photosynthesis and photosynthate transport, resulting in a local or overall carbon shortage (McDowell 2011; Sevanto *et al.* 2014; Li *et al.* 2020). Hydraulic failure and carbon starvation may also contribute to tree death caused by ageing or by trunk borer pests, as these processes also affect water transport and carbon metabolism (Frank *et al.* 2014; Wiley *et al.* 2016). In northwest China and the Loess Plateau region, low precipitation and its uneven seasonal distribution are the main obstacles for the growth and survival of trees (Guo *et al.* 2011). Meanwhile, damage caused by *H. hippophaecolus* is frequent in sea-buckthorn. Thus, a study about the mechanisms by which trunk borer pests interact with ageing trees under drought might provide new insights into the current debate about the mechanism of drought-induced tree mortality, particularly as drought and insect outbreaks may often interact to kill trees (Paine *et al.* 1997; Hubbard *et al.* 2013; Anderegg *et al.* 2015; Wiley *et al.* 2016).

Trees can repair cavitation embolism through the mechanisms of root pressure, osmotic regulation and cambium growth (Brodersen *et al.* 2010), but there is still much debate about the existence of short-term repair under tension (novel refilling) (Choat *et al.* 2019). A growing consensus suggests that embolism inevitably occurs under severe drought, and embolism repair might be possible under declining tension and strongly reduced transpiration (Secchi *et al.* 2021). Despite several repair models, it has been agreed that parenchyma cells closed to xylem play an important role in embolism repair for the provision of an osmotic potential gradient, water and energy (Chitarra *et al.* 2014; Knipfer *et al.* 2016; Secchi *et al.* 2021). Whether the restoration of hydraulic function can take place determines whether trees can quickly recover from drought stress (Skelton *et al.* 2017; Klein *et al.* 2018). Our previous studies found that elderly sea-buckthorn grew weaker and suffered higher embolism than young trees, especially when it was seriously damaged by *H. hippophaecolus*. It was speculated that this may be associated with increased embolism vulnerability or decreased embolism repair capacity, which may further influence tree growth and other physiological processes. However, the physiological mechanism of embolism repair limitation is still not fully understood.

In this study, 4-year-old trees (4y), 15-year-old normal growth trees (15yN) and 15-year-old seriously moth-damaged trees (15yH) were selected as the research objects. We focused on the following issues: (i) Growth characteristics of branches, leaves and roots of different sea-buckthorn types. (ii) The effects of tree ageing and *H. hippophaecolus* damage on hydraulics and non-structural carbohydrate (NSC) balance of sea-buckthorn, and the physiological mechanism of tree death caused by tree ageing and moth damage. (iii) Limiting factors of xylem embolism repair in sea-buckthorn damaged by ageing and sea-buckthorn moth.

## Materials and Methods

### Plant material and field sites

**Figure 1.**
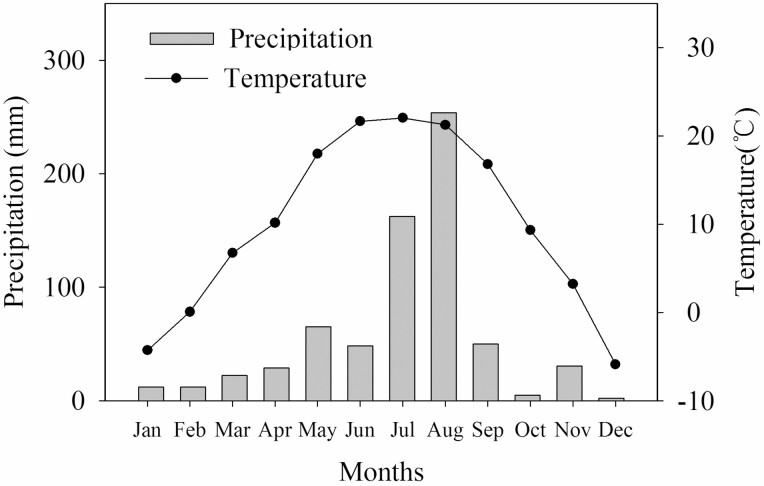
Temperature and precipitation in the experimental area in 2020.

This study was carried out in the experimental nursery of the College of Forestry, Shanxi Agricultural University (112°34ʹ58″E, 37°25ʹ47″N), Taigu District, Shanxi Province, China. The elevation of the field site is about 780 m, located in the eastern margin of the Loess Plateau and having a warm temperate continental climate. The annual average sunshine duration is 2530.8 h, the annual average temperature is 8–10 °C and the annual average rainfall is 437.4 mm, varying seasonally under the influence of the monsoon climate. Seasonal drought usually occurs in the early growing season (before July), and rainfall is concentrated in July–August. Thus, the growing season of the area is divided into wet and dry conditions. The temperature and precipitation data of the experimental year are shown in Fig. 1.

Sea-buckthorn (*H. rhamnoides* ‘Shenqiuhong’) was used in this study. The 4-year-old sea-buckthorn was transplanted in spring of 2017, and the 15-year-old sea-buckthorn was transplanted in spring of 2006, with row spacing of 2 m and planting distance of 1.5 m. Some of the 15-year-old sea-buckthorn trees declined or even died entirely due to damage caused by ageing or the sea-buckthorn moth (*H. hippophaecolus*). In this study, sea-buckthorn trees with normal growth and no signs of diseases and insects were regarded as normal 15-year-old or 4-year-old sea-buckthorns. Those with obvious signs of insects at the trunk base were identified as 15-year-old insect-damaged sea-buckthorns. Twelve individuals of each type were selected for measurements. After the completion of the experiment, the trees were dug up to verify the extent of insect damage. The severity of the trunk base damage and taproot cavities served as the standard to verify the degree of moth damage.

### Growth of branches and fine roots

The growth indexes of current-year twigs and fine roots of the trees studied were measured in August 2020. The current-year branches were sampled at the middle-upper and outer part of the canopy, and the leaves were sampled at the same position. The leaf area was measured with a leaf area meter (LI-3100C, Li-Cor Inc., Lincoln, NE, USA), and the leaves were dried in an oven to obtain the leaf mass. The leaf mass per area was calculated by dividing the leaf mass by the leaf area.

The fine root density was measured using the digging method. At each of 0–20 cm, 20–40 cm and 40–60 cm horizontal distances from the trunk, and each of 0–20 cm, 20–40 cm and 40–60 cm in depth, a 20 cm × 20 cm × 20 cm cubic soil block was dug out, giving nine soil blocks in total per tree. The roots were carefully collected from the soil, and fine roots < 2 mm in diameter were scanned with an EPSON Perfection V700 scanner. The scanning images were analysed with Win Rhizo software. The root density was calculated by dividing the total root length by the volume of the soil taken.

### Water potential, percentage loss of conductivity and vulnerability curve measurements

In the middle of June and in the middle of August 2020, 1-year-old twigs growing in the middle-upper and outer part of the canopy were sampled to determine water potential using a water potential meter (PMS 600D, Albany, NY, USA); six plants in each group were measured. The predawn water potential (Ψ_pd_) was measured at 5:00–5:30 before sunrise, and the midday water potential (Ψ_md_) was measured at 12:00–14:00. Twigs (10 cm long) were cut off at the top of branches and immediately measured.

The timing of the predawn and midday measurement of percentage loss of conductivity (PLC) in branches was the same as that for water potential, and the branch (about 1 cm in diameter) collection site was also the same as for water potential measurements. The branches were cut 30–40 cm from the top of the plants under water, the ends were immersed in water and the branches were brought back to the laboratory for determination within 30 min. A segment about 3 cm long was cut off from the material under water and both ends were trimmed with a new blade prior to measurement. The PLC and maximum specific water conductivity were obtained according to the method of Dai *et al.* (2018). A low-pressure flow meter (LPFM) was used for these measurements. The determination solution was 0.025 mol·L^−1^ KCl solution, which was prepared with distilled water filtered through a 0.22-µm micromembrane and pumped by vacuum pump. Firstly, the KCl solution was pushed through the branch segment with a pressure difference of 4 kPa, and the outflow liquid was collected in a high-precision balance. A computer connected to the balance calculated the flow rate using LPFM software, and the initial flow rate *K*_i_ (hydraulic conductivity, kg·m·s^−1^·MPa^−1^) was recorded after the reading in the software stabilized. The embolism was removed by high-pressure flushing with the same KCl solution at 0.175 MPa for about 5 min until the flow rate stopped rising. Then the maximum flow rate *K*_max_ was obtained in the same way as *K*_i_. Percentage loss of conductivity was calculated as (1 − *K*_i_/*K*_max_) × 100 %. Branch-specific hydraulic conductivity (*K*_s_) and leaf-specific hydraulic conductivity (*K*_l_) were calculated as hydraulic conductivity divided by xylem cross-sectional area and total leaf area distal to the segment, respectively (Liu *et al.* 2018).

The vulnerability curves (VCs) were measured using the air injection method (Tyree *et al.* 1998; Wang *et al.* 2014). The branches were sampled in the same way as for PLC measurement at predawn. The maximum vessel length was measured using the air method (Wang *et al.* 2014). The base of the branch was trimmed with a new blade and attached to a plastic pipe, and the other end of the plastic pipe was injected with compressed air at a pressure of 0.15 MPa. The top of the branch was immersed in water and segments (2 cm long) were sequentially cut off from the top to observe whether bubbles emerged. If bubbles were observed, the length of the remaining branch was the maximum vessel length (18 cm). For VC measurement, a segment with the maximum vessel length + pressure neck length (9 cm) was then cut off and both ends were trimmed with a new blade under water. The segment within the pressure neck was debarked, then the embolism was removed using the same method as for PLC measurement for 20–30 min. The maximum flow rate and subsequent percentage loss of conductivity (*D*) were obtained using an LPFM as for the PLC measurement. Then the pressure neck was gradually pressured to get a series of *D* corresponding to each pressure (*P*) until *D* was about 90 %. Vulnerability curves were plotted as *D* versus *P* for six branches. These curves were fitted to the Weibull equation (Cai and Tyree 2010):


$$\begin{array}{l} \frac{D}{100}=\;1-exp\left[-{\left(\frac{T}{b}\right)}^{c}\right] \end{array}$$

where *b* and *c* are fitting constants.

### Application of the 2,3,5-triphenyl tetrazolium chloride method to determine xylem cell viability

The cell viability index (CVI) was determined using the tetrazolium reduction method (Achchige *et al.* 2021). Reduction of 2,3,5-triphenyl tetrazolium chloride (TTC) was used to measure cell viability. The remaining branches sampled for predawn PLC measurement were used for CVI measurement. The branch was debarked, and cross-sections (ideally unicellular or bicellular layers) were obtained from the middle of the branch with a slicer (LEICA RM 2135, Germany). Slices were placed into 0.05 mol·L^−1^ potassium phosphate buffer solutions of 7.2 pH and kept at room temperature (20 °C). For measurement, the slices were transferred to vials containing 1.5 mL of 0.8 % TTC solution prepared in 0.05 M potassium phosphate buffer at pH 7.4. The slices were permeated in vacuum for six cycles of 15 s each and kept in darkness for 20–22 h at room temperature (20 °C). Then the slices were removed from the TTC solution and washed two to three times with deionized water, blotted with filter paper and transferred to a centrifuge tube containing 4 mL of 95 % ethyl alcohol for formazan extraction for 12–16 h. After centrifugation at 3000 rpm for 5 min to obtain the supernatant, the absorbance value at 485 nm was measured. The macerated tissue in each centrifuge tube was dried at 70 °C for 12 h and weighed. The formazan concentration was determined from the standard curve, and the mass of formazan was expressed in dry weight. Cell viability index was calculated by dividing the formazan concentration of a treated sample by the mean formazan concentration of 4-year-old samples. Thus, CVI = formazan concentration of the treated sample/mean formazan concentration of the 4-year-old sample.

### Microstructural determination

The size and location of branches used for vessel microstructural measurement were the same as those for PLC measurement. Samples were collected in August 2020, and six branches from different trees were collected for each type of tree. Branch samples were cut into several transverse sections and observed and photographed under a microscope to determine the vessel diameter and density (Davis *et al.* 1999). For a transverse section, early wood of current-year xylem of the growth ring and three visual fields were selected for measurement. The number of vessels measured for each type of sample was >100. To determine vessel density (number of vessels per unit branch transverse section area; No mm^−2^), six areas of each type of sample were selected for measurement.

### Gas exchange index measurement

The gas exchange index was measured in late June and late August in 2020. A Li-6400 portable photosynthesis apparatus (Li-Cor Inc., Lincoln, NE, USA) with a standard chamber equipped with a blue-red light source was used for the measurement at 9:00–11:00 am on sunny days. The ambient CO_2_ concentration was maintained at 410 μmol·mol^−1^, and photosynthetic effective radiation intensity was set at 1500·μmol·m^−2^·s^−1^. The gas exchange indexes, including maximum net photosynthetic rate (*A*_n_), stomatal conductance (*g*_s_) and transpiration rate (Tr), were collected. These measurements were carried out on the same dates as leaf water potential and PLC were measured.

### NSC determination

Leaf, branch and root samples with the same diameters as those for PLC measurement were collected in late June and late August in 2020. The samples were dried at 70 °C for 48 h. All dried samples were ground and sieved through a 100-mesh screen, and the powder was used for the determination of soluble sugar (SS) and starch (St) content using the anthrone method (Hanson and Moller 1975). A sample of 0.1 g of the powder was put into a 10-mL centrifuge tube, and 5 mL of 80 % alcohol (volume ratio) was added. Then the mixture was incubated in a water bath at 80 °C for 30 min, during which it was continuously shaken and mixed. After centrifugation at 3500 rpm for 10 min, the supernatant was transferred to another 10-mL centrifuge tube. The above steps were repeated twice with 80 % alcohol extraction for the remaining precipitation, and the supernatant was transferred to the same centrifuge tube. Then, 10 mg activated carbon was added to the supernatant for decolorization for 30 min, and the supernatant was filtered into a 100-mL volumetric flask. Soluble sugars were obtained. After reaching constant volume, 2 mL of the solution was transferred into a 10-mL centrifuge tube. Then 5 mL of anthrone sulphate reagent (0.2 g anthrone was dissolved in 100 mL concentrated sulphuric acid, prepared and used at any time) was added, mixed well and the mixture placed in a boiling water bath for 10 min. After cooling, the absorbance value at 620 nm was measured. Soluble sugar concentration was calculated from the standard curve.

Two millilitres of distilled water were added to the remaining precipitate after the extraction of SS, and boiled for 15 min. After cooling, 2 mL of 9.2 mol·L^−1^ perchloric acid solution was added for degradation into SS for 15 min, during which the mixture was continuously shaken and mixed. After centrifugation at 4000 rpm for 10 min, the supernatant was transferred to a 100-mL volumetric flask. The precipitate was then degraded with 2 mL 4.6 mol·L^−1^ perchloric acid solution and the above steps repeated twice. The supernatant was transferred into a volumetric flask. Then the precipitate was rinsed two to three times with 5–6 mL distilled water. Soluble sugars were obtained. The SS concentration was determined as described above.

All variables used in the analysis are shown in Table 1, and the symbols will be used later.

**Table 1.** Symbol and unit for the variables measured in the study.

Variables	Unit	Symbol
4-year-old sea-buckthorn trees	—	4y
15-year-old normal growth trees	—	15yN
15-year-old seriously moth-damaged trees	—	15yH
Predawn water potential	MPa	Ψ_pd_
Midday water potential	MPa	Ψ_md_
Percentage loss of conductivity	—	PLC
Branch-specific hydraulic conductivity	kg·m^−1^·s^−1^·MPa^−1^	*K*_s_
Leaf-specific hydraulic conductivity	kg·m^−1^·s^−1^·MPa^−1^	*K*_l_
Vulnerability curve	—	VC
Vulnerability to embolism	MPa	P_50_
Cell viability index	—	CVI
Net photosynthetic rate	μmol CO_2_·m^−2^·s^−1^	*A*_n_
Stomatal conductance	mol HO_2_·m^−2^·s^−1^	*g*_s_
Transpiration rate	mmol HO_2_·m^−2^·s^−1^	Tr
Non-structural carbohydrate	—	NSC
Soluble sugar	—	SS
Starch	—	St

The symbol ‘—’ represents no unit.

### Statistical analyses

All values were calculated as mean ± standard error (SE). One-way ANOVA was applied to test differences in parameters for the dry and wet seasons. Sample sizes for all measurements were five to six replicates. A difference was considered significant at *α* = 0.05. The statistical analyses were all performed using SPSS 22.

## Results

### Tree growth and fine root density

The height and basal diameter of 15yN and 15yH trees were significantly larger than that of 4y trees; however, the growth of annual branches in 4y trees was significantly higher than that of 15yH and 15yN trees. The length of annual branches of 15yN and 15yH trees was only 49.7 % and 30.2 % as long as that of 4y trees, and the basal diameter of annual branches of 15yN and 15yH trees was only 52.3 % and 43.2 % as long as that of 4y trees, respectively. Also, the leaf area and leaf mass per area of 4y trees were significantly larger than that of 15yN and 15yH trees, with the leaf area of 15yH trees being only 22.7 % of that of 4y trees (Table 2).

**Table 2.** Tree growth of different types of sea-buckthorn.

	4y	15yN	15yH
Height (m)	2.60 ± 0.08b	4.07 ± 0.22a	4.17 ± 0.11a
Basal diameter (cm)	5.45 ± 0.23b	9.85 ± 0.63a	9.55 ± 0.69a
Length of annual branch (cm)	15.90 ± 1.31a	7.90 ± 1.20b	4.80 ± 0.69c
Basal diameter of annual branch (cm)	2.20 ± 0.13a	1.15 ± 0.08b	0.95 ± 0.20b
Leaf area (cm^2^)	2.38 ± 0.11a	1.20 ± 0.08b	0.54 ± 0.03c
Leaf mass per area (g·cm^−2^)	111.20 ± 2.16a	100.39 ± 1.38b	88.30 ± 1.67c

Means ± standard error (*n* = 6) are shown, and different letters refer to significant difference at *P* < 0.05.

Regarding fine root density, 4y trees had a higher root density in the 0–20 cm horizontal/0–20 cm deep zone, and 15yN trees had a higher density in the 0–20 cm horizontal/20–40 cm and 40–60 cm deep zones. Comparing 15yH with 15yN trees, the fine root density of the former was significantly lower than that of 15yN trees in all measured areas (Table 3).

**Table 3.** The root length density of different types of sea-buckthorn.

		Horizontal direction								
Vertical direction			0–20 cm		20–40 cm			40–60 cm		
		4y	15yN	15yH	4y	15yN	15yH	4y	15yN	15yH
	0–20 cm	824 ± 66a	388 ± 34b	195 ± 10c	754 ± 68a	535 ± 29a	211 ± 19b	307 ± 24b	601 ± 70a	189 ± 10c
	20–40 cm	287 ± 17b	425 ± 13a	142 ± 16c	258 ± 29a	148 ± 8b	142 ± 16b	250 ± 23a	216 ± 25a	47 ± 3b
	40–60 cm	95 ± 10b	138 ± 9a	117 ± 8ab	97 ± 10a	122 ± 13a	36 ± 4b	108 ± 5a	93 ± 3a	34 ± 5b

Means ± standard error (*n* = 3) are shown, and different letters refer to significant difference at *P* < 0.05.

### Vessel characteristics and vulnerability to embolism

**Figure 2.**
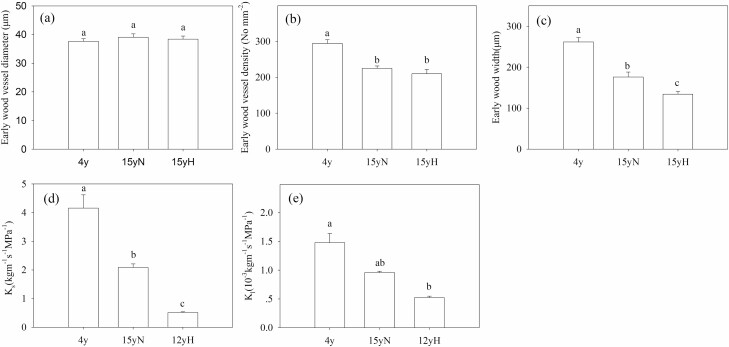
Branch xylem vessel characteristics and hydraulic conductivity of different types of sea-buckthorn. (A) Early wood diameter; (B) vessel density; (C) early wood width; (D) branch-specific hydraulic conductivity (*K*_s_); (E) leaf-specific hydraulic conductivity (*K*_l_). The number of vessels measured per section varied between 60 and 100, and three sections were observed for each xylem sample. The replicates for *K*_s_ and *K*_l_ were six (*n* = 6). Means ± standard error are shown, and different letters refer to significant difference at *P* < 0.05.

**Figure 3.**
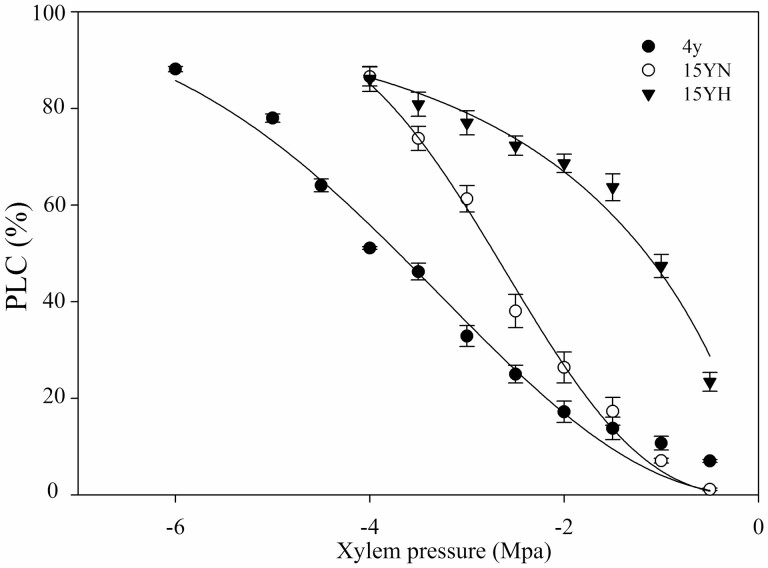
Branch embolism vulnerability curves of different types of sea-buckthorn. Means ± standard error (*n* = 6) are shown.

There was no significant difference in the diameter of early wood vessels among the three tree types. The early wood vessel density of 4y trees was significantly higher than that of 15yN and 15yH trees. There were significant differences in the width of early wood vessels, and the order from largest to smallest was 4y, 15yN and 15yH trees. Also, there were significant differences in branch-specific hydraulic conductivity between types of tree, with the conductivity of 15yN and 15yH trees being only 50.2 % and 12.3 % of that of 4y trees, respectively (Fig. 2). The order of embolism vulnerability of branches from smallest to largest was 4y, 15yN and 15yH, with their P_50_ (xylem water potential corresponding to 50 % of PLC) being −3.69 MPa, −2.71 MPa and −1.15 MPa, respectively (Fig. 3).

### Water potential

**Figure 4.**
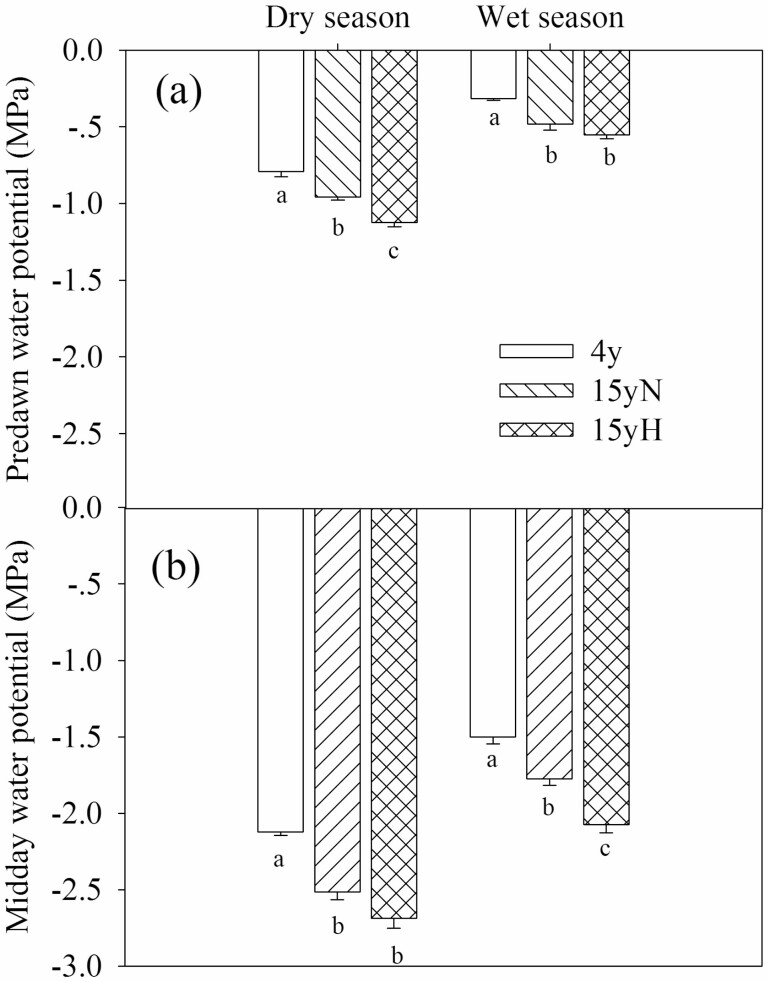
Predawn and midday water potential of different types of sea-buckthorn. (A) predawn water potential; (B) midday water potential. Means ± standard error (*n* = 6) are shown, and different letters refer to significant difference in the same season at *P* < 0.05.

In the dry season, there were significant differences in Ψ_pd_ among the three types of tree, and the order from largest to smallest was 4y, 15yN and 15yH. The Ψ_md_ of 15yN and 15yH trees was significantly lower than that of 4y trees, with no significant difference between 15yN and 15yH. In the wet season, the Ψ_pd_ of 15yN and 15yH trees was significantly lower than that of 4y trees. There were significant differences in Ψ_md_ among the three types, and the order from highest to lowest was 4y, 15yN and 15yH. Both Ψ_pd_ and Ψ_md_ were significantly lower in the dry season than in the wet season for each group (Fig. 4).

### Percentage loss of conductivity

**Figure 5.**
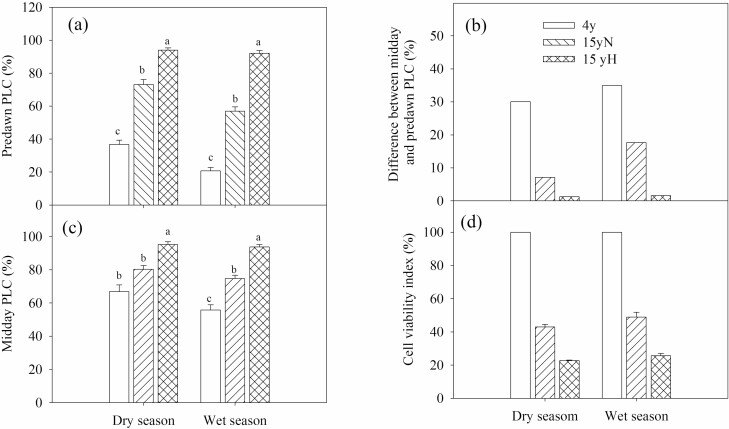
Percentage loss of conductivity and cell viability index of different types of sea-buckthorn. (A) Predawn PLC; (B) midday PLC; (C) difference between midday and predawn PLC; (D) cell viability index. Means ± standard error (*n* = 6) are shown, and different letters refer to significant difference in the same season at *P* < 0.05.

In both the dry and wet seasons, there were significant differences in the predawn PLC among the three types, and the order from highest to lowest was 15yH, 15yN and 4y. The predawn PLC of 4y trees was 36.8 % and 20.8 %, the predawn PLC of 15yN trees was 73.2 % and 57.0 % and that of 15yH trees was 94.1 % and 92.1 % in the dry and wet season, respectively. The order of midday PLC from highest to lowest was also 15yH, 15yN and 4y. The midday PLC of 4y trees was 66.9 % and 55.7 %, and that of 15yH trees was 95.3 % and 93.8 % in the dry and wet season, respectively (Fig. 5).

The order of PLC variation from midday to predawn was 4y > 15yN > 15yH, with that of 15yH trees being near to zero, while the PLC of 4y trees varied by 30.1 % and 35.0 % in the dry and wet season, respectively. There were significant differences in CVI among 4y, 15yN and 15yH in both seasons, from highest to lowest in 4y, 15yN and 15yH trees (Fig. 5).

### Photosynthetic rate and stomatal conductance

**Figure 6.**
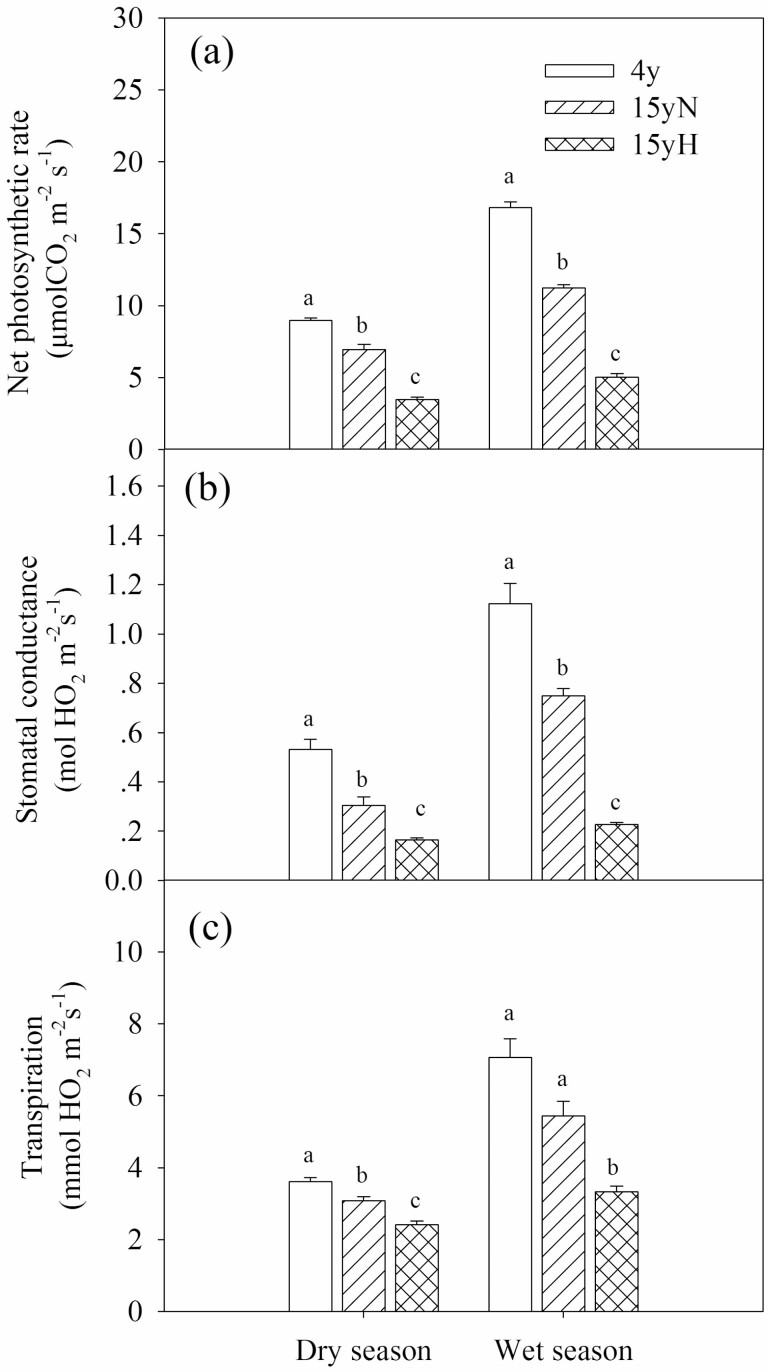
Photosynthetic rate, stomatal conductance and transpiration rate of different types of sea-buckthorn. Means ± standard error (*n* = 6) are shown, and different letters refer to significant difference in the same season at *P* < 0.05.

In both the dry and wet season, there were significant differences in *A*_n_, *g*_s_ and Tr among the three types of tree except for Tr between 4y and 15yN in the wet season, and the order from highest to lowest was 4y, 15yN and 15yH (Fig. 6).

### NSC content

**Figure 7.**
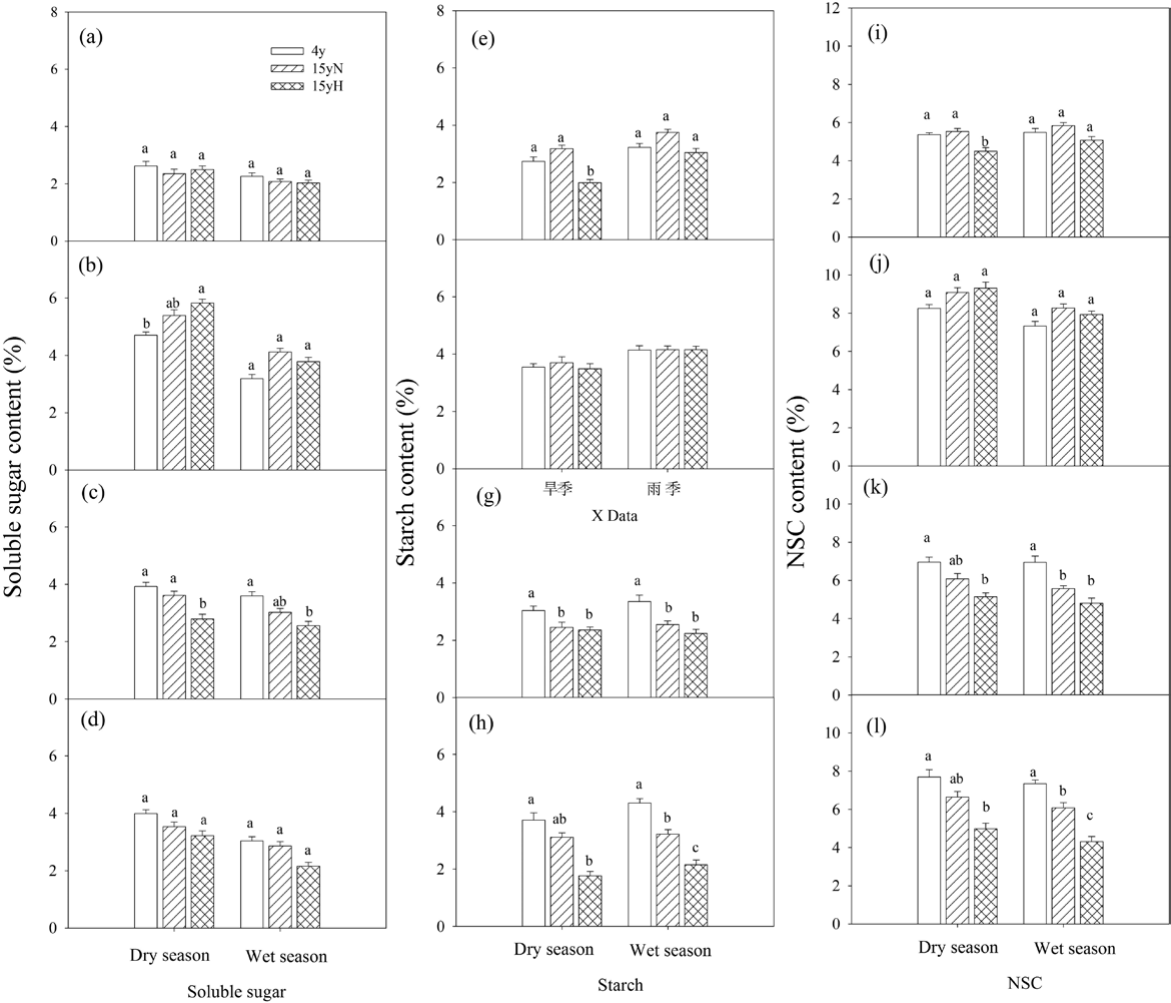
Soluble sugar, starch and NSC content in branches and roots of different types of sea-buckthorn. (A) Branch xylem soluble sugar content; (B) branch phloem soluble sugar content; (C) root xylem soluble sugar content; (D) root phloem soluble sugar content; (E) branch xylem starch content; (F) branch phloem starch content; (G) root xylem starch content; (H) root phloem starch content; (I) branch xylem NSC content; (J) branch phloem NSC content; (K) root xylem NSC content; (L) root phloem NSC content. Means ± standard error (*n* = 6) are shown, and different letters refer to significant difference in the same season at *P* < 0.05.

There were no significant differences in branch xylem SS among 4y, 15yN and 15yH trees in the dry or wet season. The branch xylem St and NSC content of 4y and 15yN trees were significantly higher than that of 15yH in the dry season, but not significantly different in the wet season. The branch phloem SS of 4y trees was significantly lower than that of 15yH trees in the dry season, and the root xylem SS of 4y trees was significantly lower than that of 15yH trees in both seasons. However, the root xylem St of 4y trees was significantly higher than that of 15yN and 15yH trees in both seasons, and the root xylem NSC content of 4y trees was significantly higher than that of 15yH trees. There was no significant difference in root phloem SS between seasons in any group, but there were significant differences in root phloem St and NSC content, with the order from highest to lowest being 4y, 15yN and 15yH (Fig. 7).

## Discussion

### Effects of tree ageing and insect damage on hydraulic structure

Wood moth feeding can impair the outer xylem and cambium of the trunk base and coarse roots, destroying water-conducting tissues, and thus affecting water transport from root to leaf (Ueda *et al.* 2005). In this study, the Ψ_pd_ and Ψ_md_ in both seasons were lowest in 15yH trees, which suggests that moth damage affects the water use of trees. The Ψ_pd_ and Ψ_md_ of 15yN were also significantly lower than that of 4y trees, indicating that ageing is also an important factor influencing sea-buckthorn water status. Tree water status influences physiological processes to a great extent, especially the growth of trees (McDowell 2011). Fifteen-year-old sea-buckthorn showed obvious growth decline, as reflected by the results of this study.

Sea-buckthorn is a ring-porous tree species, so current-year early wood vessels play a major role in hydraulic conductivity (Umebayashi *et al.* 2016). Although there was no significant difference in vessel diameter, the vessel density of the two types of 15-year-old tree decreased significantly to about 200 No mm^−2^ compared to 294 No mm^−2^ in 4y trees, and the early wood width decreased to 176 and 134 μm for 15yN and 15yH trees compared to 261 μm in 4y trees, respectively. According to the Hagen–Poiseuille law (Cruiziat *et al.* 2002), hydraulic conductivity is positively correlated with the number of vessels and the fourth power of vessel diameter, so the differences in vessel density and early wood width may be responsible for the differences in hydraulic conductivity. In both seasons, the PLC from highest to lowest was 15yH > 15yN > 4y. The midday PLC of 15yN reached more than 80 %, and that of 15yH reached over 90 %. Embolism was commonly observed during drought stress (Sevanto *et al.* 2014; Choat *et al.* 2019; Hammond *et al.* 2019), but in the wet season the older trees still suffered severe embolism. The PLC induced by cavitation embolism is mainly determined by the water potential, vulnerability to embolism and the capacity for embolism repair (Patrizia *et al.* 2015; Hammond *et al.* 2019; Skelton *et al.* 2021). In this study, the severe PLC may be the result of the combined effects of these factors.

Vulnerability to embolism was reflected in VC, consistent with the results of PLC. The vulnerability to embolism has been assessed in a large number of tree species, and P_50_ was considered to be an index related to drought resistance (Choat *et al.* 2012; Anderegg *et al.* 2016). Cavitation resistance was correlated with wood anatomy, e.g. the structure of intervessel pits and the thickness of the vessel wall (Lens *et al.* 2011; Christman *et al.* 2012; Bouche *et al.* 2014). Although the microstructural changes associated with cavitation resistance were not clear in the current study, the cavitation resistance of the old trees was weakened during ageing or pest feeding, which made them susceptible to cavitation embolism. We also found that the PLC variation from midday to predawn was highest in 4y trees, while that in 15yN and 15yH trees was small, even less than 5 %, indicating that the limited embolism repair capacity was also an important reason for the larger PLC.

It is widely believed that embolism can be repaired; however, the specific mechanism of embolism repair has not been fully revealed. Embolism repair under positive pressure has been widely recognized, but root pressure plays a small role in trees and cambium growth takes a long time, so ‘novel refilling’ may be the main mechanism of short-term xylem embolism repair (Brodribb *et al.* 2010; Patrizia *et al.* 2015; Schenk *et al.* 2021). It is widely believed that ‘novel refilling’ mainly relies on the generation of local positive pressures via osmotic mechanisms, created by solute release from vessel-associated parenchyma into the embolized conduits (Secchi and Zwieniecki 2012). Therefore, the activity of xylem parenchyma cells plays an important role in embolism repair (Chitarra *et al.* 2014; Secchi *et al.* 2021). In this study, the cell viability of 15yN and 15yH trees decreased significantly to ~40 % and ~20 %, respectively, relative to the 4y trees in both seasons, which may affect the process of embolism repair. Day-by-day accumulation of embolism during prolonged drought might lead to extensive xylem dysfunction.

### Effects of tree ageing and insect damage on carbon distribution

In this study, gas exchange variables suggested that water transport affected photosynthesis. The effect of water stress on photosynthesis has been extensively studied (Woodruff and Meinzer 2011; Hartmann 2015; Bloemen *et al.* 2016). Plants reduce water loss by closing their stomata during drought, and stomatal closure also reduces the CO_2_ diffusion in the leaf, resulting in reduced photosynthetic carbon uptake and photosynthetic products (Hartmann 2015). Water stress also affects carbon transport, so photosynthates and stored carbon cannot be transported to the desired location (Sevanto *et al.* 2014). Meanwhile, wood moth damaged the phloem of the stem base and coarse roots, inevitably influencing phloem carbon transport (Lahr and Krokene 2013; Wiley *et al.* 2016). This affected carbon uptake, transport and distribution, thus changing the whole or partial NSC accumulation and storage, and further affecting the growth of trees. In this study, the St concentration in the branch xylem of 15yH trees decreased significantly in the dry season, but its phloem SS was significantly higher than that of 4y trees, which may be due to the influence on carbon transport from phloem to xylem in moth-eaten sea-buckthorn.

As described above, the transport of SS from phloem to xylem is involved in the maintenance of osmotic pressure required for embolism repair, so this transport may be closely related to the activity of xylem parenchyma cells. Therefore, we speculated that the decreased cell viability would affect the radial transport of photosynthate and further embolism repair. In addition, NSC was the source of defensive substances, metabolizable energy and maintenance of osmotic pressure (McDowell 2011; Hartmann 2015). During long-term drought stress, plants only relied on stored NSC, which necessarily led to insufficient NSC, thus affecting these functions, especially hydraulic function (Hartmann 2015). The differences in the NSC of roots were significant between the 4y and 15yH trees in both seasons and between 4y and 15yN trees in the wet season, in both xylem and phloem, indicating that the ability to transport NSC to the roots was reduced in 15yN and 15yH trees. The tree ageing and pest damage in 15yH trees severely affected the NSC supply to the root, which would further affect the growth and absorption function of the roots and aggravate the degree of water stress.

Overall, the increased branch PLC of the older sea-buckthorn restricted photosynthesis and carbon distribution. And the hydraulics of the older sea-buckthorn infested by wood moth were further restricted, leading to significantly reduced root NSC supply and constrained NSC radial transport from phloem to xylem, which in turn restricted root growth, root absorption function and branch embolism repair. The increase in PLC in the aged sea-buckthorn was related to the increase in vulnerability to embolism and the limitations to embolism repair caused by the decreased xylem cell viability.

## Conclusions

Fifteen-year-old trees, either exposed to ageing or pests, had poorer hydraulics and carbon metabolism than normal 4-year-old trees, in both seasons. Significantly lower water potential, more severe embolism, lower photosynthesis and reduced root NSC led to poor water status and insufficient carbon supply, which inevitably affected plant growth and other physiological processes. Higher vulnerability to embolism and lower cell viability in turn further worsened the water status and carbon balance, which impacted the plants’ ability to cope with drought stress. So they suffered from hydraulic dysfunction and carbon shortage. For 15-year-old trees exposed to pests, in addition to ageing, almost all indexes were worse than in the ageing trees, which further aggravated the decline of the trees.

## Supporting Information

The following additional information is available in the online version of this article—

plac051_suppl_Supplementary_Data

## Data Availability

The data used in this study are available in the online supplementary data of this article.

## Notes

**Form & Function. Chief Editor:** Kate McCulloh
